# Analytical performances of the Xpert MTB/RIF assay using stool specimens to improve the diagnosis of pulmonary tuberculosis in Burkina Faso, a tuberculosis endemic country

**DOI:** 10.1371/journal.pone.0288671

**Published:** 2023-07-31

**Authors:** Odilon D. Kaboré, Anselme Millogo, Bintou Sanogo, Emile Birba, Armel Poda, Boubacar Nacro, Olivier Marcy, Sylvain Godreuil, Abdoul-Salam Ouédraogo

**Affiliations:** 1 Department of Bacteriology and Virology, Souro Sanou University Hospital, Bobo Dioulasso, Burkina Faso; 2 Superior Institute of Health Sciences, NAZI BONI University, Bobo-Dioulasso, Burkina Faso; 3 Laboratory of Emerging and Re-emerging Pathogens, School of Health Sciences Nazi Boni University, Bobo Dioulasso, Burkina Faso; 4 Département de Pédiatrie du Centre Hospitalier Universitaire Sourô Sanou, Bobo-Dioulasso, Burkina Faso; 5 Service de Pneumologie-Phtisiologie du Centre Hospitalier Universitaire Sourô Sanou, Bobo-Dioulasso, Burkina Faso; 6 Service des Maladies Infectieuses du Centre Hospitalier Universitaire Sourô Sanou, Bobo-Dioulasso, Burkina Faso; 7 Bordeaux Population Health Research Center Inserm U1219, University of Bordeaux, Bordeaux, France; 8 Laboratoire de Bactériologie, CHU de Montpellier, MIVEGEC (IRD, CNRS, Université de Montpellier), Montpellier, France; 9 Muraz Center, Bobo Dioulasso, Burkina Faso; Shandong Public Health Clinical Center: Shandong Provincial Chest Hospital, CHINA

## Abstract

Timely diagnosis of Pulmonary Tuberculosis (PTB) is associated with good prognosis, but remains difficult in primary healthcare facilities and particularly in children and patients living with HIV. The aim of this study was to compare the GeneXpert ® MTB/RIF assay (Xpert) performed using a stool sample (3–5 g) and using the first Respiratory Tract Sample (RTS; *i*.*e*., sputum, bronchoalveolar or gastric aspirate; as normally done) concomitantly collected from 119 patients with suspected PTB to improve PTB diagnosis in Burkina Faso, a high tuberculosis burden country with limited resources. Overall, microbiological, microscopic and molecular analysis of the 119 first RTS and 119 stool specimens led to *Mycobacterium tuberculosis* complex detection in 28 patients (23 positive RTS cultures and 5 negative RTS cultures-RTS Xpert positive). When using the 28 clinical confirmed cases as reference standard, the sensitivities of the stool-based and RTS-based Xpert assays were not different (24/28, 85.7%, *versus* 26/28, 92.86%; *p* > 0.30), and 22 results were fully concordant. Considering the first RTS culture as the gold standard, the sensitivities of the stool-based and RTS-based Xpert assays to detect PTB in patients with positive RTS culture were 100% (23/23) and 91.3% (21/23), respectively (*p* >0.05). The stool-based Xpert assay specificity for excluding PTB was 99% (95/96) (compared with 95%, 91/96, when using RTS) and its negative and positive predictive values were 100% (95/95) and 96% (23/24), respectively. Compared with the 23 positive RTS cultures, the incremental yield rates of the RTS-based and stool-based Xpert assays were 4.2% (5/119) and 0.84% (1/119), respectively. Overall, our findings support using the stool-based Xpert assay as an alternative method for earlier PTB diagnosis, when RTS are difficult to obtain.

## Introduction

Tuberculosis (TB), an infectious disease caused by *Mycobacterium tuberculosis* complex, is an important contributor to the overall disease burden. In 2020, despite the ambitious global goals set by the World Health Organization (WHO) and the United Nations to reduce TB epidemic global burden, TB was diagnosed in approximately 5.8 million individuals and caused 1.5 million deaths (including children and patients living with HIV). Moreover, multidrug-resistant TB is an increasing concern worldwide and directly threatens disease-control efforts in many countries. These figures for 2020 were considered an underestimation because the SARS-CoV-2 pandemic led to an underdiagnosis of TB cases, and this is likely to increase TB spread [1].

In addition, timely diagnosis of Pulmonary TB (PTB), especially in children and in adults living with HIV and prostrated by the disease, remains difficult when using standard sputum-based assays in primary healthcare facilities, resulting in low diagnostic yields. The diagnosis in these populations poses serious challenges due to: *i)* the paucibacillary nature of sputum samples; *ii)* the invasive and painful nature of the procedures (bronchoalveolar or gastric lavage) used to the collect high-quality Respiratory Tract Samples (RTS), and *iii)* the need of qualified professionals to perform these procedures who are not usually available at primary healthcare centers in resource-constrained and high-burden settings [2–4]. As adults and infants swallow intermittently low amounts of *M*. *tuberculosis* complex, we hypothesized that bacterial load in stool could become progressively more concentrated during the intestinal transit time (18–24 hours), and thus such *M*. *tuberculosis* complex could be easily detected if they reached the Xpert MTB/RIF assay lowest limit of detection (131 colony-forming units [CFU]/mL) [5]. The Xpert MTB/RIF (Xpert herewith; the first point-of care assay for TB endorsed by WHO in 2010) and Xpert MTB/RIF Ultra (the newest version of the Xpert assay with a limit of detection of 15.6 CFU/mL, designed to overcome the low Xpert assay sensitivity in patients with paucibacillary disease) assays are major innovations for TB diagnosis. Indeed, these tests simultaneously detect *M*. *tuberculosis* complex and rifampin resistance within two hours of sampling and with minimal hands-on technical time, and are therefore suitable for point-of-care testing at the primary healthcare level. The WHO has already published recommendations for its wide effective use with stool samples for the diagnosis of pediatric PTB [6–8].

In the present report, we share our first experience of implementing the Xpert MTB/RIF assay by evaluating its performance using a large sample (at least 3–5 g) of stool as an alternative to culture and to the RTS-based Xpert assay in patients with suspected PTB.

## Patients and methods

### Ethical approval

This study was authorized by the local ethics committee of the Centre Hospitalier Universitaire Sourô Sanou, 01 BP 676 Bobo-Dioulasso 01, Burkina Faso (2016/0027/MS/RHBS/DRS). Written informed consent was obtained from all participants and the parents or guardians of the <18-year-old children included in the study. The questionnaires did not mention the patients’ name to ensure their anonymity.

### Study type, duration, and enrolled patients

This comparative and prospective cross-sectional study was carried out from January to July 2016 (*i*.*e*., 7 months). All consecutive clinical specimens from patients with suspected pulmonary TB sent to the hospital laboratory for bacteriological confirmation were included.

### Clinical specimens

Clinical specimens were fecal samples (one sample per patient) and RTS (sputum, bronchial and alveolar washing). In <10-year-old children, gastric aspiration was systematically performed. In adults who could expectorate easily, two sputum specimens were systematically collected according to our TB program control guidelines, but only the first sputum sample was analyzed with the Xpert assay (due to the limited number of available cartridges). Microscopy analysis and culture were performed for all RTS and fecal samples.

### Microbiological procedures

#### Specimen digestion and decontamination

RTS and fecal samples (3–5 g) were decontaminated following the modified Petroff’s method for *M*. *tuberculosis* complex culture [9], as recommended by the Burkina Faso National Tuberculosis Control Program. Briefly, an equal volume of 4% NaOH/0.2% KOH solution was added to the 50-mL sterile and leak-proof tube containing the sample, followed by homogenization in a shaker at room temperature for 40 min. Then, samples were neutralized with sterile phosphate buffer (PBS) and centrifuged at 1,509 g at room temperature for 20 min. Pellets were used to prepare the smears, inoculate Löwenstein-Jensen (LJ) medium, and for the Xpert assay. Moreover, 2 mL of each pellet was stored at -80° C.

### Microscopic analysis

Smears prepared from RTS and stool pellets were air-dried and heat-fixed. This was followed by auramine O and Ziehl-Neelsen (ZN) staining, according to the Burkina Faso National Tuberculosis Control Program guidelines, and in line with the international guidelines [10]. Positive and negative control smears were prepared from known positive and negative samples and stained in parallel with the study samples.

### Bacteriological culture

For culture, three drops of each RTS/fecal pellet were inoculated on LJ medium and incubated at 37°C. In the first week, cultures were monitored daily to check for contamination. Then, they were inspected twice per week for up to 8 weeks to verify the presence of colonies. Colonies were systematically stained with ZN staining to confirm the presence of acid-fast bacteria (AFB) before *M*. *tuberculosis* complex identification (see below). A culture was considered negative, if no colony was detected after 8 weeks of incubation. The number of culture failures included the number of contaminated cultures. In case of contaminated culture, the pellet stored at -80°C was decontaminated again and a new culture was started.

### *M*. *tuberculosis* complex identification

In positive cultures, the SD Bioline Ag MPT64 immunochromatographic test (Standard Diagnostics Inc., South Korea D Bioline, South Korea) was used to rapidly detect the MPT 64 protein in *M*. *tuberculosis* complex isolates with a mouse anti-MPT 64 monoclonal antibody, as described elsewhere [11].

#### Gene Xpert® MTB/RIF assay

The Xpert assay was performed using the RTS pellets according to the manufacturer’s instructions [7]. Results were automatically generated within 2 hours and reported as *M*. *tuberculosis* complex detected, not detected, or indeterminate, and rifampicin-susceptible or -resistant. *M*. *tuberculosis* complex detection is based on the amplification of two *rpoB* gene regions, and rifampicin susceptibility status is determined based on the difference (>3.5 amplification cycles) for any probe. Stool pellets were resuspended in 2 mL PBS and the supernatant was analyzed with the Xpert assay following the manufacturer’s instructions using a 2:1 ratio of Xpert reagent to sample. If the Xpert assay result was “error” or “invalid”, the sample was tested again until the result was negative or positive.

#### Data processing and analysis

Data were described using means and standard deviations (SD) and Microsoft Excel 2013 for Window and the Epi info7 software. Sociodemographic characteristics (mean age, sex ratio) were described. The percentage of confirmed PTB cases was calculated, and then univariate analysis was used to compare the percentages of confirmed TB cases in function of age, sex, and HIV status. Data between groups were compared with the Pearson Chi square test if normally distributed, otherwise with the Fisher’s exact test. The significance threshold of 5% was used for all statistical tests. The sensitivity, specificity, positive and negative predictive values (PPV and NPV) of the Xpert assays with RTS and stool samples for pulmonary TB diagnosis were calculated using the RTS culture as gold standard, the RTS smear results, and the number of confirmed TB cases.

#### Pulmonary tuberculosis diagnosis

In this study, PTB was diagnosed in the presence of (i) a positive culture for *M*. *tuberculosis* complex and/or (ii) of a positive Xpert assay result for the first RTS. The Xpert assay diagnostic performance was calculated relative to the LJ culture results and to a composite reference standard (positive Xpert assay and/or culture).

## Results

### Demographic and clinical characteristics of enrolled patients

During the study period, 119 patients were enrolled. Their male/ female sex ratio was 1.05 (61 men and 58 women) and the mean age was 31 ± 18 years (range: 7 months to 78 years). The 31–40 years age group was the most represented (n = 30; 25.21%), followed by <10-year-old children (n = 21; 17.65%). In this population, 68 (57.14%) patients were HIV-positive, 17 (14.29%) were HIV-negative, and the other 34 patients did not know their HIV status. In the HIV-positive group (n = 68), CD4^+^ T-cell count was ≤ 200 cells/mm^3^ in 20 (29.41%) patients, ≥ 200 cells/mm^3^ in 27 (39.70%) patients, and unknown in 21(30.90%) patients **(S1 Table)**. Most patients were hospitalized in the departments of infectious and tropical diseases (40.34%), pediatrics (31.09%) and pulmonology (20.17%). All other patients came from the emergency services (3.36%), otorhinolaryngology department (0.84%), or were outpatients (4.20%).

**Fig 1:**
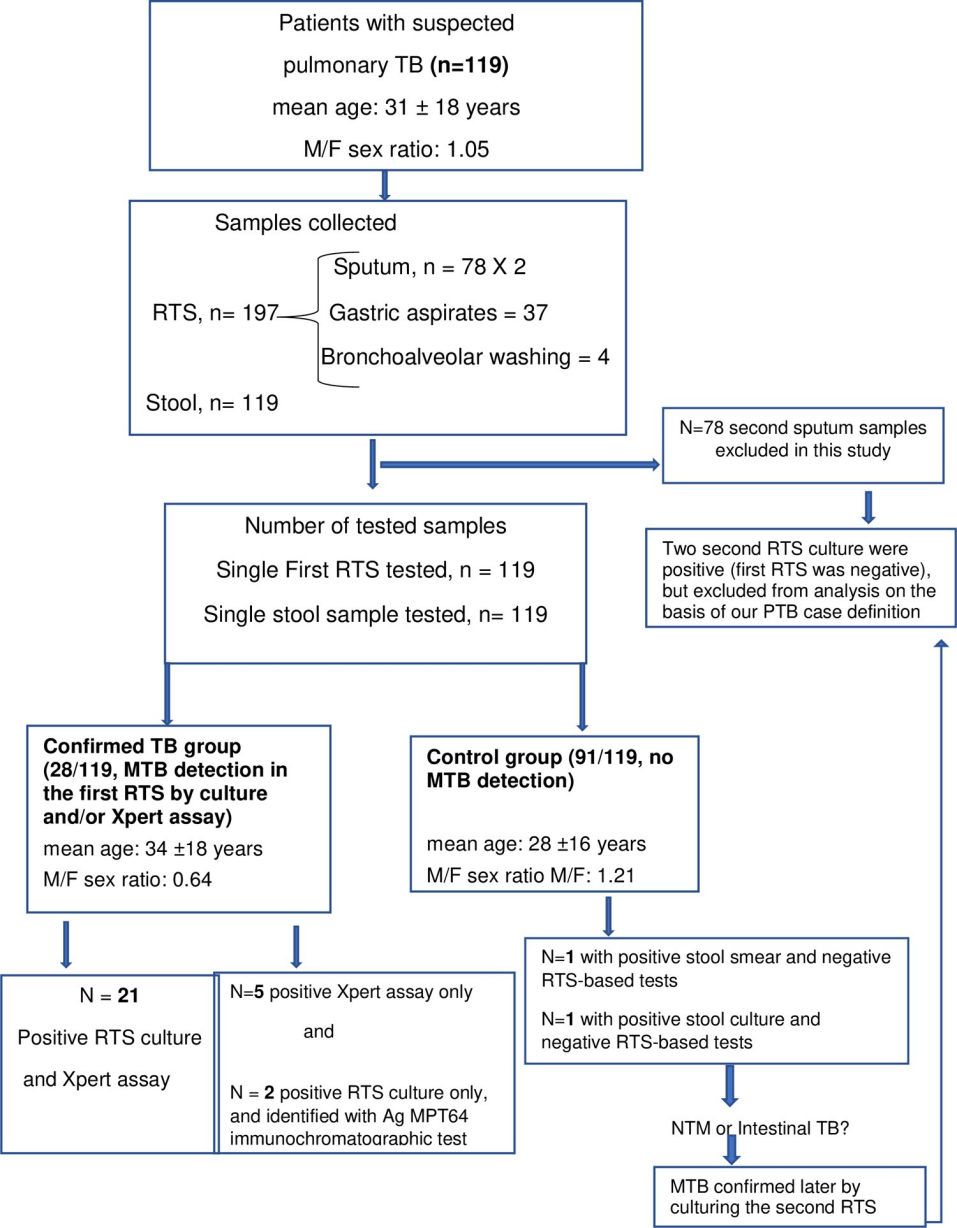
Study flow diagram. The Xpert assay was performed using the first RTS and the 3–5 g of stool sample from the 119 recruited participants. **Abbreviations:** TB, tuberculosis; MTB, *Mycobacterium tuberculosis* complex; RTS, Respiratory Tract Sample.

Only one RTS could be obtained from 41 patients (34.4%): i) 37 gastric aspirates from 37 pediatric patients and ii) 4 bronchial washings from 4 prostrated adults. Two consecutive sputum samples were obtained from 78 patients (65.6%), but the second sample was not evaluated in this study because the number of available Xpert cartridges was not sufficient to test both samples in all patients. The second sample was used only for microscopy analysis and culture. Concomitantly, one stool specimen was collected from all 119 patients and was analyzed with the Xpert assay in parallel with the first RTS (**Fig 1**).

### Patients with confirmed pulmonary tuberculosis

Analysis of the first RTS allowed confirming the diagnosis of PTB diagnosis in 28/119 (23.53%) patients: in 21 patients both first RTS culture and Xpert assay were positive, in 2 patients only the culture, and in 5 patients only the Xpert assay. The mean age of these 28 patients (n = 11 men and n = 17 women) was 34 ± 18 years, and 18 were HIV-positive (64%), 3 were HIV-negative and 7 had unknown HIV status. The TB group included two <10-year-old children among whom one was HIV-positive. Pulmonary TB was excluded in 91 patients (control group). Their age was 28 ± 16 years, 50 were men and 41 were women. However, microbiological/microscopical analysis of the second RTS, available for 78 (65.6%) patients, identified two additional cases of PTB that were not included in the present analysis (**Fig 1**).

### Contamination rate of bacteriological cultures

Overall, 11 (9.2%) RTS cultures and 73 (61.34%) stool cultures were contaminated. Among these contaminated cultures, one (1/11) RTS and ten (10/73) stool cultures were AFB-positive by microscopy. A second decontamination of the pellet of these AFB-positive samples (stored at -80° C) was performed after the first culture failure, but the culture was again contaminated or *M*. *tuberculosis* complex did not grow (**Table 1**).

**Table 1:** Total number of RTS and stool culture failures.

Culture and sample type	RTS			Stool samples		
	Smear+	2^nd^ decontam		Smear+	2^nd^ decontam		
		C +	C -		C+	C-	
**Contaminated cultures**	1/11	0	1	0/73	0	10	
**Non-contaminated cultures**	16/108	**-**	**-**	5/46	**-**	**-**	
Total	17/119	**-**	**-**	15/119	**-**	**-**	

C+: positive culture; C-: negative culture; 2^nd^ decontam: Second decontamination

### *M*. *tuberculosis* complex detection rate, comparison of the results obtained with RTS and stool samples

AFB were observed in 17/119 (14.3%) first RTS and in 15/119 (12.6%) stool smears. In 12 of the 17 patients with AFB-positive RTS, the stool smear also was AFB-positive. The first RTS culture was positive for *M*. *tuberculosis* complex in 23/119 patients (19.3%) and the stool culture was positive in 9/119 patients (7.6%). Both RTS and stool cultures were positive in 5 patients (21.73% of 23). The Xpert assay detected *M*. *tuberculosis* complex DNA in 26/119 RTS (21.8%) and in 24/119 (20.2%) stool samples (in both samples for 22/26 patients, 84.61%) (**Table 2**). The diagnostic accuracy (microscopy analysis, culture, and Xpert assay) of stool samples, compared with the first RTS, is summarized in **Table 3**. In two of the four patients with only RTS-based Xpert positive results, stool smear and culture also were positive (a 27-year-old woman and a 45-year-old man).

**Table 2:** Comparison of the results of stool and RTS smear, culture, and Xpert assay to detect the presence of *M*. *tuberculosis* complex.

**Smear (n = 119)**	Stool smear positive (n = 15)	Stool smear negative (n = 104)
RTS smear positive (n = 17)	12	5
RTS smear negative (n = 102)	3	99
**Culture (n = 119)**	Stool culture positive (n = 9)	Stool culture negative (n = 110)
RTS culture positive (n = 23)	5	18
RTS culture negative (n = 96)	4	92
**Xpert assay (n = 119)**	Stool Xpert positive (n = 24)	Stool Xpert negative (n = 95)
RTS Xpert positive (n = 26)	22	4
RTS Xpert negative (n = 93)	2	91

**Table 3:** *M*. *tuberculosis* complex detection rate and concordance using RTS and stool samples and different diagnostic methods in the 28 patients with confirmed pulmonary TB (microbiological culture).

RTS positive results (28/119)	MTB detection rate in stool (%)		
	M+	C+	GX +
**M+, C+ and GX + (n = 16)**	12 (75%)	3 (18.8%)	16 (100%)
**M-, C+, and GX + (n = 5)**	0	2 (40%)	5 (100%)
**M-, C+, and GX- (n = 2)**	0	0	2 (100%)
**M+, C-, and GX+ (n = 1)**	0	1	1
**M -, C-, and GX+ (n = 4)**	2 (50%)	2(50%)	0
**M -, C-, and GX- (n = 0)**	1^a^	0	0
**M -, C-, and GX- (n = 0)**	0	1^b^	0

M+: positive smear; M-: negative smear; C+: positive culture; C-: negative culture; GX+: positive Xpert MTB/RIF assay; GX-: negative Xpert MTB/RIF assay

^a^ In this patient with more 30 AFB/microscopic field, pulmonary TB was confirmed later by culturing the second RTS.

^b^ In this patient, pulmonary TB was confirmed later by culturing the second RTS.

In the 15 patients with positive stool smear **(Table 3)**, the stool-based Xpert assay was negative in 3 patients among whom 2 had a positive RTS-based Xpert assay. Thus, the stool-based Xpert assay displayed a detection rate of 80% (12/15) when using at least 3 g of stool. The three negative stool samples had positive stool smear (>30 AFB/microscopic field) and were from prostrated HIV-positive patients. Therefore, they were tested again (using several dilutions of the remaining stored stool sample) with new cartridges to see if these AFB were not nontuberculous mycobacteria (NTM). In this second analysis, *M*. *tuberculosis* DNA was detected.

### Sensitivity, specificity, NPV and PPV of stool-based smear, culture and Xpert assay

RTS culture is the gold standard for active PTB diagnosis confirmation. Therefore, the diagnostic accuracy of stool-based microscopy, culture and Xpert assay to detect PTB was compared to the RTS culture results (**Table 4**). This comparison showed the good performance of the stool-based Xpert assay. Conversely, the sensitivity of stool smear and culture was low.

**Table 4:** Sensitivity, specificity, and positive and negative predictive values of the three methods using stool samples compared with the 23 positive RTS cultures (gold standard).

Stool	Sputum culture			Sensitivity (%)	Specificity (%)	PPV (%)	NPV (%)
		Positive	Negative				
**Smear**	Positive	12	3	52.2	96.9	80.0	89.4
	Negative	11	93				
**Culture**	Positive	5	4	21.7	95.8	55.6	83.6
	Negative	18	92				
**Xpert**	Positive	23	1	100	99.0	95.8	100
	Negative	0	95				

### Incremental yield of the stool-based and RTS-based Xpert assays for pulmonary TB detection

TB bacteria are intermittently secreted from the respiratory tract, leading to RTS-based Xpert negative results (<131 CFU/ml) in patients with paucibacillary TB. To test whether swallowed bacteria may become concentrated in stool (thus above the Xpert lower limit of detection, >131 CFU/ml) during the intestinal transit times (18–24 hours), the stool-based and RTS-based Xpert assays were compared using as reference the RTS culture results (**Table 5**) and the 28 confirmed cases of PTB (**Table 6**). This comparison highlighted that among the 23 samples with RTS culture positive results, two were found positive by the stool-based and not by the RTS-based Xpert assay.

**Table 5:** Diagnostic performance of the stool-based and RTS-based Xpert MTB/RIF assays compared with the 23 positive RTS cultures (gold standard).

Sample type/Performance	RTS Xpert [n = 119]	Stool Xpert [n = 119]
**Sensitivity %**	91.3% (21/23)	100% (23/23)
**Specificity %**	95.0% (91/96)	99% (95/96)
**PPV %**	80.8% (21/26)	96% (23/24)
**NPV %**	97.8% (91/93)	100% (95/95)

PPV: Positive Predictive Value, NPV: Negative Predictive Value

**Table 6:** Clinical sensitivity, specificity, and predictive values of the stool-based and RTS-based Xpert assay compared with the 28 confirmed cases of pulmonary TB.

Sample type/Performance	RTS Xpert	Stool Xpert
**Sensitivity %**	93% (26/28)	85.7% (24/28)
**Specificity %**	100% (91/91)	100% (91/91)
**PPV %**	100% (26/26)	100% (24/24)
**NPV %**	97.8% (91/93)	95.8% (95/95)

PPV: Positive Predictive Value, NPV: Negative Predictive Value

### Detection of rifampin resistance

Among the 28 confirmed cases of PTB, the Xpert assay detected 18 rifampicin-susceptible and 9 rifampicin-resistant *M*. *tuberculosis* complex isolates. Results were comparable for rifampicin susceptibility when using the RTS-based and the stool-based Xpert assays, except in one patient in whom sputum testing yielded rifampicin susceptibility whereas rifampicin resistance was undetermined when examining stools.

## Discussion

This study supports the utility of stool as an alternative sample for PTB diagnosis. Indeed, the Xpert assay using at least 3 g of decontaminated and concentrated stool sample gave results that were fully concordant with RTS culture, the gold standard (100% of sensitivity), and led to an incremental yield of 0.84% (1/119) compared with RTS culture. These findings highlight the potential value of the stool-based Xpert assay for early PTB diagnosis in primary healthcare settings where microbiological culture facilities are not available. Similarly, El Kechine et *al*. previously reported 100% of sensitivity for real-time PCR detection of IS6110 (using RTS culture as gold standard) [12], and Dubale et *al*. [13] also reported 100% of sensitivity of the stool-based Xpert assay using culture as reference standard. In addition, Seble et *al*. found 100% of sensitivity, although they compared the stool-based Xpert assay to RTS smear positivity, which is not the gold standard [14]. Our sensitivity (100%) was higher than what was reported in previous studies: 94.8% for adults [15], 88.9% for children [16], and 86% using IS6110 testing by PCR in fecal samples from adults with pulmonary TB [17], compared with RTS culture. These differences in sensitivity can be due to the larger quantity (at least 3 g) of the stool samples tested in our study. Indeed, Banada et *al*. [18], and Rahman et *al* [15], stated that the Xpert assay sensitivity was 84% when using 1.2 g of stool sample in pediatric patients [18], and 94.8% when using 2 g of stool samples in adult patients [15]. However, one limitation of our 100% sensitivity for stool-based Xpert assays was the high RTS culture contamination rates of 9.2%, leading to a sensitivity of 82.21% (23 RTS positive samples /28 PTB confirmed cases) of RTS culture instead of 100% (28/28), thus limiting its value as reference standard. That is why we observed the incremental yields of the RTS-based and stool-based Xpert assays of 4.2% (5/119) and 0.84% (1/119) relative to the number of positive RTS cultures (n = 23). Our RTS cultures contamination rates, greater than the recommended threshold of 5% for laboratories that receive freshly collected RTS [10], may be due to the sample that take several days to reach our laboratory (oral microflora continue to grow and reduce the decontamination effectiveness). This is consistent to those who reported a contamination rates of 5 to 10% for such samples [19].

In the group of 28 patients with confirmed PTB (positive culture and/or Xpert assay), the stool-based Xpert assay was positive in patients with positive RTS smear (17/17,100% sensitivity) and also with negative RTS smear (7/11, 63.6% sensitivity). Overall, its clinical sensitivity accuracy was 85.7%. Similarly, Kokuto et *al*. [20] reported a clinical sensitivity of 85.7% (48/56) using stool samples from adult with pulmonary TB. Moreover, in our group of patients with confirmed pulmonary TB, the sensitivity of the stool-based and RTS-based Xpert assays for the clinical diagnosis of pulmonary TB were comparable (24/28, 85.7% and 26/28, 92.86%, respectively; *p* > 0.30), and 22/28 results were concordant. Recently, in China, the Xpert Ultra assay was equally sensitive using stool and gastric samples (85.4%, 41/48) from children with confirmed pulmonary TB [21]. This finding supports our results on the utility of stool samples as an alternative to RTS samples.

In four patients, pulmonary TB was diagnosed based only on the positivity of the RTS-based Xpert assay (negative stool-based Xpert assay). For two of these patients, the stool smear and culture were positive (**Table 3**), suggesting that inhibitors present in stool samples can inhibit the DNA Taq polymerase [18]. On the other hand, in two patients with “unconfirmed pulmonary TB”, based on the negative RTS results, one was stool culture-positive and stool-based Xpert assay-negative, and the other was stool smear-positive but stool-culture and stool-based Xpert assay-negative. As NTM has been previously detected in patients’ stools [22], and *M*. *tuberculosis* complex has been detected in patients with intestinal TB without lung infection [23], these stool samples were tested again to rule out NTM presence using diluted stool pellets. This new analysis allowed detecting *M*. *tuberculosis* complex DNA and suggests that PCR inhibitors in concentrated stool samples can reduce the PCR performance [18]. Furthermore, the detection of >30 AFB/microscopic field in stool smears from prostrated HIV-positive patients indicates that a low inoculum of AFB excreted intermittently and swallowed becomes more concentrated in stool during the transit time (18–24 hours) and can be easily detected [24]. It is a well-known that HIV alters the course of TB infection. Indeed, 24% to 61% HIV-positive individuals have a higher rate of sputum smear-negative PTB (paucibacillary PTB) because they are less likely to have cavitary lesions due to the impairment of granuloma formation [25, 26]. The likelihood of disseminated TB (miliary) increases with higher HIV viral loads and CD4 count (≤ 200 cells/mm^3^) [27]. In this group of patients, *Mycobacterium tuberculosis* complex is more excreted in their feces and the use of stool sample for PTB diagnosis has been found to be useful in this vulnerable group [28, 29]. This is supported in the latest recommendation of the WHO on the use of stool Xpert MTB/RIF assay for PTB diagnosis since 2021 [30]. Conversely, in these paucibacillary patients, *M*. *tuberculosis* presence in RTS is erratic, thus requiring consecutive RTS sampling using invasive and painful procedures to confirm the diagnosis. In the two patients with “unconfirmed pulmonary TB”, the diagnosis was subsequently confirmed by serial sputum culture (positive second RTS).

Considering stool culture sensitivity, the low confirmation rate of pulmonary TB should be considered with the increased processing requirements and higher culture contamination rates (**Table 1**), in agreement with previous studies [31, 32]. Indeed, the contamination rate was higher with stool specimens than RTS samples (73/119 *versus* 11/119), explaining the low sensitivity of stool culture [22]. The modified Petroff’s decontamination method was suitable for RTS but not for stool decontamination [9]. The major drawback of stool culture is the need of stringent decontamination/concentration techniques to prevent the overgrowth of gut microbiota. It would be most effective to combine 1% chlorhexidine with the method described by Kent and Kubica, as reported elsewhere [12, 33].

The strengths of this study include the testing of a high amount of stool sample (at least 3) that led to a good correlation of the stool-based Xpert and RTS smear and culture (gold standard) results. The limitations include the high contamination rate of RTS cultures that created an imperfect reference standard for active PTB diagnosis, the PCR inhibitors and error/invalid results (possibly due to filter clogging in the Xpert cartridge) with the stool-based Xpert assay using a large volume concentrated of fecal sample. Recently, Lounnas and colleagues [34] developed a centrifuge-free processing method for the stool-based Xpert Ultra assay using a low quantity (0.5 g) of stool samples from pediatric patients. This method has a sensitivity of 70%. Nevertheless, optimized protocols for the Xpert assay using larger volumes of stool to increase sensitivity and with <10% of invalid/error results are needed [18]. Another limitation of our study was the limited number of available Xpert cartridges that did allow testing only the first RTS sample and one stool sample. Marcy et *al*. showed that when different samples (RTS and stool) are concomitantly analyzed with the Xpert assay, sensitivity increases by 10%, thus improving the chances of TB diagnosis [35]. Nevertheless, our results are comparable to what reported by Marcy et *al*. statements and with those of studies in patients co-infected with HIV [36] and in children [18]. Overall, our findings indicate that in primary healthcare settings, the stool-based Xpert assay with a single large and concentrated stool sample (at least 3 g) could improve the rapid diagnosis and allow early treatment of PTB while waiting for RTS culture confirmation in a tertiary hospital. The marketing of the Xpert MTB/RIF Ultra cartridges requires new studies to develop a suitable stool processing method and to assess the performance using such stool samples to improve the detection of PTB in our resource-constrained setting.

## Supporting information

S1 Table Distribution of age, type of Respiratory Tract Sample, HIV-status and CD4 T-cell count against detection rate of *MTC* in sputum and stool among confirmed PTB cases (n = 28). (PDF)
