# Supplementary material for: Analytical performances of the Xpert MTB/RIF assay using stool specimens to improve the diagnosis of pulmonary tuberculosis in Burkina Faso, a tuberculosis endemic country
Source: PLoS One. 2023 Jul 31;18(7):e0288671. doi: 10.1371/journal.pone.0288671 (PMC10389731; doi:10.1371/journal.pone.0288671)
Supplement: S1 Table — (PDF) [file pone.0288671.s001.pdf]

S1 Table. Distribution of age, type of Respiratory Tract Sample, HIV-status and CD4 T-cell count against detection rate of *MTC* in sputum and stool among confirmed PTB cases (n=28)

Specimen types, Demographic and clinical Characteristics	RTS specimens			Stool specimens		
**Enrolment group (n=119)**<br>mean age: 31 ± 18 years<br>sex-ratio M/F: 61/58<br>**Control group (91/119)**<br>mean age: 28 ±16 years<br>sex-ratio M/F: 50/41<br>**Case group (28/119)**<br>mean age: 34 ±18 years<br>sex-ratio M/F: 11 /17	AFB	Culture	Xpert	AFB	Culture	Xpert
**Total of detection**	**17**	**23**	**26**	**15**	**9**	**24**
**Age and type of RTS**						
Pediatric patients with gastric aspirate (years ≤12 (2/37)	1	2	1	2	1	2
Prostrated patients With Brochoalveolar Washing (years ≥ 12 (0/4))	0	0	0	0	0	0
Patients with sputum (years ≥12 (26/98)	16	21	25	13	8	22
**HIV-status**						
**HIV Positive (18/68)**	**12**	**16**	**17**	**10**	**6**	**17**
CD4≤200 (4/20)	3	4	4	4	3	4
CD4≥200 (5/27)	1	3	5	1	0	4
Unknow CD4 (9/21)	8	9	8	5	3	9
**HIV Negative=3/17**	**2**	**3**	**2**	**1**	**1**	**3**
**Unknow HIVstatus (7/34)**	**3**	**4**	**7**	**4**	**2**	**4**
